# Supplementary material for: Research priorities of caregivers and individuals with dementia with Lewy bodies: An interview study
Source: PLoS One. 2020 Oct 7;15(10):e0239279. doi: 10.1371/journal.pone.0239279 (PMC7540843; doi:10.1371/journal.pone.0239279)
Supplement: S1 File — (DOCX) [file pone.0239279.s002.docx]

**S1 File.** Semi-structured interview guide for **“**Research Priorities of Caregivers and Individuals with Dementia with Lewy Bodies: An Interview Study”

**Welcome:** Thank you for participating in this interview. We are interested in learning more about what is most important to you as you or your loved one lives with dementia with Lewy bodies. We are interested in two related things:

(1) the clinical care you/your loved one receive(s) for dementia with Lewy bodies and whether there are ways we can make it better, and

(2) what kind of research you think should be done for people with dementia with Lewy bodies and their families.

We want to know your overall thoughts and also your thoughts on specific topics. We will interview 25 people with DLB and 25 caregivers of people with DLB for this study.

We will be audio recording the discussion so that we review the interview later. There are no right or wrong answers, just different points of view. You may choose not to answer any question and we can stop at any time. What questions do you have?

**Demographics:**

Before we start, I have a few questions that will help us understand the background of the people participating in the interviews:

1. Are you a person diagnosed with DLB or a caregiver of someone with DLB?

2. What is your age (in decades)? <30? 30-39? 40-49? 50-59? 60-69? 70-79? ≥80?

3. What is your gender?

4. What is your race?

5. What is your highest level of education?

**Semi-Structured Interview:**

First, we’re going to discuss your *clinical care*.

1. What parts of your/your loved one’s care for dementia with Lewy bodies have been most helpful? (Responses expected to relate to patient care +/- caregiver experience)

2. What needs do you and/or your loved one have that are not currently being met by the clinic? (Responses expected to relate to patient care +/- caregiver experience)

3. What DLB symptoms hasn’t your/your loved one’s doctor addressed?

4. If you could make one change that would make the Clinic better, what would you change?

Now we’re going to discuss your opinions about what is most important to *research* in dementia with Lewy bodies.

1. For what topics related to dementia with Lewy bodies (DLB) do you wish there were more research?

- Possible prompt: Are their unmet needs in your care (e.g. how you were diagnosed, how you are treated) that could prompt a new research study?

Now I am going to ask you about a series of issues related to DLB. Please tell me if more research is needed for that topic, why, and what suggestions you have.

2. What DLB *symptoms* do you hope research can better address?

- Possible prompt: What symptoms affect your (or your loved one’s) quality of life the most?

3. What day-to-day life challenges should research address?

4. What topics relating to caregiving or family life in DLB should research address?

Follow-up questions:

(4a) What topics relating to a caregiver’s journey could research address?

(4b) When people have difficulty with memory and thinking, often family members have to help make decisions or take over decision making completely. What topics relating to decision-making could research address?

(4c) Families often need support and education. What topics regarding support or type of education should research address?

5. What are your thoughts regarding research to see if DLB can be diagnosed earlier?

6. When we study brain problems like DLB we can focus on different parts of the disease, such as:

- how much abnormal protein or shrinkage there is in the brain

- people’s symptoms like thinking, movement, or mood problems

- global issues like quality of life or daily function.

(6a) How important do you think it is focus on each of these?

(6b) What areas of focus are most important to you and why?

(6c) Are there any areas of research that I didn’t mention that you believe are equally or more important to address?

7. If you had $1000 to spend on DLB research, how would you divide that money between different research topics?

**Ending Question:**

We are coming to the end of the interview. Of all the things we discussed today, what to you is the most important?

**Closing:**

Thank you so much for your willingness to share your thoughts with us. Your opinions today will help us as we plan ways to improve our dementia with Lewy body clinic and as we plan upcoming research on DLB. When we have finished all our interviews, we will be developing a summary of what people said. Would like to receive a copy? (If yes, ask for preferred address, either email or postal.)

Thank you again.
